# Prevalence and pattern of renal complications of uterine fibroids in a Teaching Hospital in Nigeria

**DOI:** 10.4314/ahs.v24i4.21

**Published:** 2024-12

**Authors:** Mumuni Amisu, Joy Chionuma, Ayoola Odeyemi, Odewale Odetayo

**Affiliations:** 1 Lagos State University College of Medicine, Medicine; 2 Lagos State University College of Medicine, Obstetrics and Gynaecology; 3 Lagos State University Teaching Hospital, Medicine; 4 Lagos State University Teaching Hospital, obstetrics and Gynaecology

**Keywords:** Fibroids, uropathy, nephropathy, uterine leiomyomata

## Abstract

**Background:**

Symptomatic uterine fibroids causing obstructive nephropathy were thought to be uncommon, but recent prevalence studies showed otherwise. The aim of the study is to determine the prevalence of obstructive uropathy and nephropathy in patients with uterine leiomyoma.

**Methodology:**

The study was a hospital-based cross-sectional retrospective study of 114 patients diagnosed with symptomatic uterine fibroids seen in the gynecology clinic of Lagos State University Teaching Hospital, Ikeja from February 2021 to June 2022.

**Results:**

The overall prevalence of obstructive uropathy and nephropathy was 22.8% and 10.5% respectively. The average level of serum creatinine was 2.2 ± 1.9mg/dl with a creatinine range of 1.8 to 6.7mg dl. The average of the GFR among patients was 29.3 ± 13.4ml/min/1.73m^2^ with a range of 9 – 54ml/min/1.73m^2^. There is a positive association between uterine size and uropathy and subjects with uropathy are more likely to develop nephropathy. (CI 95% 3.281 - 108.670).

**Conclusion:**

This study showed that prevalence of obstructive nephropathy in the patients with uterine leiomyomata is 10.5%. Uterine size and myoma diameter were positively associated with development of obstructive nephropathy

## Introduction

Fibroid or uterine leiomyoma is a smooth muscle tumor of the uterus with some fibrous tissue infiltration. It is the commonest benign neoplasm that occurs. The prevalence of uterine myomas ranges from 20-25% depending on the age, race or ethnicity, parity and type of imaging technique employed. 1 Fibroid is very common in middle age and at advancing age. Commonly documented age range is 15 to 50 years and is said to be commonest in people of black racial descent. 1,2 Fibroid is largely known to be asymptomatic but could be symptomatic in some cases when it presents with abnormal uterine bleeding (menorrhagia and intermenstrual bleeding), dysmenorrhea, and pelvic heaviness or discomfort. Notable complications include infertility and miscarriages. 2 Extra reproductive organ effects of fibroid include compressive or obstructive symptoms of urological or renal organs. For example, urology complications could be urinary retention due to urethral obstruction, uterovesical fistula, hematuria, dyspareunia, urinary incontinence or neurogenic bladder; while common renal complications are hydronephrosis, hydroureter, upper or lower urosepsis, and renal insufficiency of either acute kidney injury (AKI) or chronic kidney disease (CKD)^3^. Some authors have also documented the occurrence of hypertension in patients with bilateral hydronephrosis. The exact incidence of obstructive uropathy or nephropathy due to uterine leiomyoma is not known 4,5,6. The prevalence varies from region to region. It is said to range from 8.1 to 33.4% and this is because different researchers employ different radiological, ultrasonography or computerized tomography (CT) modalities to evaluate the uterine fibroids, especially their impacts on the urethra, ureter and the kidneys 4, 5, 6. More so, the lower incidences recorded in some works may be attributed to early presentation and management of fibroids in those climes.

The renal complications arising from the obstructive effect of fibroid on the kidney are quite devastating and challenging, especially when it results in acute or chronic kidney diseases with attendant need for renal replacement therapy. This work thus poses to identify the prevalence and pattern of renal disease in individuals with uterine leiomyomata.

The justification of the study stems from the need for early detection of patients with uterine fibroids complicated by obstructive uropathy who if left unattended could progress to obstructive nephropathy or advanced CKD.

## Methodology

This was hospital-based cross-sectional retrospective study of 114 patients with symptomatic fibroids that attended gynecology clinic of LASUTH Ikeja from Feb 2021 to June 2022. The ethical approval was obtained from the Health Research Ethical Committee (HREC) of the hospital. The inclusion criteria included patients diagnosed with symptomatic fibroids within the review period. Patients with major causes of CKD, for example, hypertension, diabetes or recurrent urosepsis and clinical or laboratory evidence of chronic kidney were excluded. The data of these patients were extracted from their clinical records which include information on demography, clinical estimation of the uterine size (approximated to fundal height in a pregnant uterus and reported as weeks of gestation), abdominopelvic scan reports showing fibroid location, number and diameter. Reports on the renal system including those that showed distortion of renal architecture, for example (hydronephrosis, hydroureter and cortical thinning) were also extracted. Results of urinalysis, urine culture and the renal function tests were also documented. Patients with eGFR < 60ml/min plus, using serum creatinine of > 1.3 mg/dl as surrogate marker or with proteinuria taken at 2 different occasions at 2 to 4 weeks intervals, were tagged as having obstructive nephropathy.

Data was entered into micro soft excel and then exported into Statistical Packages for Social Sciences (SPSS IBM) version 23.0 for further analyses. Statistical analysis used were mean, frequency distribution, suitable tables and graphs. The Chi square test determined association between variables. P < 0.05 was taken as statistically significant. Binary logistic regression was done to identify independent predictors of nephropathy.

## Results

**Fig 1:**
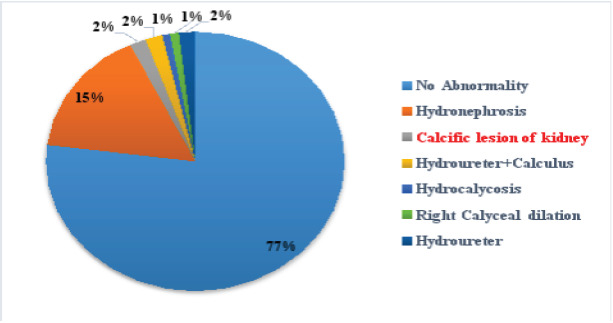
Renal and Extra renal manifestation of fibroid

A total of 114 respondents with uterine fibroids at the Lagos State University Teaching Hospital, Ikeja were included in this study. The mean age of subjects is 39.1 ± 6.8 years. More than half of the subjects 68(59.6%) were nulligravida. Most of the subjects 32(28.1%) had a uterine size (by fundal height estimation as done in a pregnant uterus) between 16/52 to 20/52 weeks of gestation on clinical palpation. One hundred and eight of the subjects (94.7%) presented with a fibroid diameter of < 5(cm) estimated by ultrasound whereas, just six (5.3%) subjects presented with a diameter of ≥ 5(cm). The average diameter of the fibroid size was 1.3±1.6 cm with a range of 0.1 to 8.4cm. A higher proportion (36.8%) of the fibroids were intramural however, thirty-one of the respondents (27.2%) had both intramural and submucosal fibroids, while (17.6%) had mainly submucosal fibroids. Figure 1 showed the pattern of renal manifestations observed in these women. The majority, 88(77.2%) of them had no associated renal impairment. Hydronephrosis was observed in 18(11.5%) of the women of which 5(4.4%) had bilateral affectation. Seven subjects 7(77.8%) had a serum creatinine level of 1.4 to 3.3mg/dl. The mean serum creatinine is 2.9 ± 1.6mg/dl, eight (66.7%) presented with proteinuria only, while 5(41.7%) had elevated serum creatinine and proteinuria. Uropathy was associated with the palpated uterine size per abdomen in this study (P= 0.001). There was, however, noassociation observed between it and the age of the woman or fibroid size. There was association between the palpated uterine size (P = 0.002), the diameter of the fibroid nodules (P =0.015) and the development of nephropathy in these women.

The logistic regression result shows that subjects with uropathy are 19 times more likely to develop nephropathy with a confidence interval of 95% 3.281–108.67. The wide confidence range may be due to relatively small sample size; however, it may also indicate that there is logical relationship between the confidence interval and the P value.

## Discussion

This study assessed the renal manifestation or changes in women with uterine fibroids and possible associations between the characteristics of the fibroid and the development of uropathy or nephropathy. A larger percentage, 52(45.6%) of women diagnosed with uterine fibroids were aged between 40 to 49 years old. This age group was slightly older than was observed in a similar study conducted in Northern Nigeria where the patients were between, 36–40 years. More so, the mean age of the women (39.1 ± 6.8 years) was comparable with the findings of Lawal Y and Yaro IB 8 (36.6 ± 1.44years) as well as in the study conducted in Nnewi (35.7 ± 6.1 years)^9^ and Ibadan (39.3 ± 9.6 years)^10^

Nulliparity has been implicated to have a strong link with the development of fibroids, and in this study, more than half (59.6%) of the women, were nulligravida. This was slightly lower than the 76% documented by Ezeama et al^9^,^11^ and Ukwenya et al (75%) 12. The average fibroid diameter of 1.3± 1.6 cm (0.1 to 8.4cm) in this study showed a similarity with the findings by Melanie Polin and Hye-Chum Hur who reported in their study that myomas size ranged from <2 cm to 12.5 cm.^13^

The location of uterine fibroid has been shown to be closely associated with the clinical manifestation of fibroid.^14^ Findings from this study reveal that more than a third, 42(36.8%) of the women had intramural fibroids. This observation is similar to that documented by Chen J et al, where a majority of the cases (68%) were intramural.^15^ usually, uterine fibroid exacts a compressive pressure on the uterus consequently resulting in urinary retention, hydroureter, hydronephrosis, and potentially post-renal kidney failure.^9^,^16^. The true incidence of hydronephrosis and renal impairment due to uterine fibroids is unknown. This is because many patients who have ultrasonography for fibroids do not have an evaluation of the ureters and kidneys. In many of these cases, the patients are asymptomatic and unless both kidneys are severely obstructed, they most often have normal values for test of renal function. Findings from this study revealed that although the majority 88(77.2%) of the subjects did not have any renal complication, there were cases of unilateral hydronephrosis 13(11.5%), bilateral hydronephrosis 5(4.4%), hydroureter 2(1.8%), calcific degeneration 2(1.8%) and hydrocalycosis 1(0.9%). This observation is similar to the findings of Horace MF and Gillian W., who reported that uterine fibroids associated with hydronephrosis accounted for 8.1% of the cases of renal impairment. Among the patients with hydronephrosis in their study, 15/31 (48.4%), had right-sided hydronephrosis while 11/31 (35.4%) had bilateral obstructive uropathy. Our study submitted a lower occurrence in these regards probably because Horace MF et al studied a larger population but our work still affirms that fibroids can result in both unilateral and bilateral hydronephrosis (uropathy).^17^ Our study showed the prevalence of uropathy to be 22.8%. This was lower than that documented by Apoku et. al as well as Idowu et al that documented a prevalence of 33 -37.1% but higher than the 11% reported by Soyebi and Awosanya.^6^,^18^,^19^,^20^

Findings from this study revealed an average level of serum creatinine of 2.9 ± 1.6mg/dl. This level was similar to what was reported by Fletcher HM et al 17, with a mean serum creatinine level of 3.31mg/dl. Bansal T, et al 21 however, reported a lower average creatinine level of 2.45mg/dl in patients with uterine fibroid. In our work, about half of the subjects 5(55.5%) presented with an eGFR of (30 – 59) ml/min/1.73m^2^ (CKD) 3 while one subject 1(11.1%) presented with an eGFR of 9.0 ml/min/1.73m^2^, in end-stage renal disease (ESRD) and is currently on hemodialysis. These outcomes lay credence to the work of Fletcher and Dawkins et al who discovered that a number of fibroid patients came down with advanced renal disease.^17^, ^25^

Actually, studies that documented prevalence of proteinuria in obstructive nephropathy caused by uterine fibroid are limited. However, our study noted 8(66%) subjects had proteinuria which may be partly due to the compressive pressure effects on the nephron. Some of the protein that could be detected include excretion of myoglobin in urine especially in patients with; color; background-color: huge uterine smooth muscle mass.

With respect to the association between the size of the uterus and uropathy we found out that subjects whose uterine size were between 25/52 to 39/52 had the highest uropathy prevalence of 41.3% and nephropathy prevalence of 21.7% respectively compared with small fibroid sizes, also incidentally found to be statistically significant (P=000I and 0.002). This study showed a relationship between uterine sizes and renal impairment. Idowu et al and Bansal et al papers were in consonants with our study^18^, ^26^. Conversely, most of the subjects with huge uterine fibroid sizes did not have expected concomitant astronomical increase in serum creatinine and proteinuria. This could be due to locations of the fibroid and the population study. However, binary logistic regression shows that subjects with uropathy are 19 times more likely to develop nephropathy with confidence interval at 95% 3.281-108.670.

## Limitations of the study

Our work is a single centre study and may not reflect the actual prevalence of obstructive nephropathy obtainable in other studies. In addition, majority of the patients could not afford necessary investigations and this affect the relatively small population size studied. MDRD was used to calculate the eGFR but not it is not the best parameter for patients in very early or advanced CKD.

## Conclusion

Our study submitted that10.5% of patients with uterine fibroid developed obstructive nephropathy in CKD stage 3. It is therefore imperative to detect this susceptible group early and treated in order to avert or retard progression to end stage renal disease (ESRD) which can be financially burdensome to manage with attendant future increased in morbidity and mortality.

## Figures and Tables

**Table 1:** Demographic characteristics of patients

Variable	Frequency N= 114	Percentage (%)
**Age Group**		
20 to 29	15	13.2
30 to 39	41	36.0
40 to 49	52	45.5
50 to 59	6	5.3
**Age range**	**23 – 54**	
**Mean ± SD**	**39.1 ± 6.8**	
**Parity**		
0	68	59.6
1	21	18.4
2	10	8.8
3	9	7.9
4	4	3.5
5	2	1.8
**Range**	**0 – 5**	
**Mean ± SD**	**0.8± 1.3**	
**Gravidity**		
Null gravida	68	59.7
Prim gravida	21	18.4
Multigravida	25	21.9

**Table 2:** Uterine size by fundal height estimation, ultrasound estimation of fibroid diameter and location of Fibroid

Variable	Frequency (N=114)	Percentage (%)
**Uterine Size (approximated to weeks of gestation)**		
10/52 to 14/52	16	14.0
16/52 to 20/52	32	28.1
21/52 to 25/52	24	21.1
26/52 to 30/52	25	21.9
31/52 to 35/52	12	10.5
36/52 and above	5	4.4
**Fibroid Diameter(cm)**		
<5	108	94.7
≥ 5	6	5..3
**Mean ± SD**	**1.3± 1.6**	
**Range**	**0.1 – 8.4**	
**Location of Fibroid**		
Mucosal	23	20.2
Serosal and mural	5	4.4
Intramural submucosal, large subserous	2	1.8
Intramural and submucosal	31	27.2
Mural	42	36.8
Serosal	7	6.1
Subserosal + polypoid	4	3.5

**Table 3:** eGFR and Proteinuria Variations in Patients with Fibroid

Variable	Frequency	Percentage (%)
**Urinalysis(proteinuria))n=l2**		
Proteinuria	8	66.7
Normal	4	33.3
**eGFR (ml/min/1.73m^2^)**	N=114	
≥60	105	92.1
<60	9	7.9
**Grading of eGFR<60**		
30 – 59	5	55.6
15 – 29	3	33.3
<15	1	11.1
**Range**	**9 – 54**	
**Mean ± SD**	**29.3 ± 13.4**	

KEY: Serum creatinine >1.3mg/dl is a surrogate marker for nephropathy using MDRD eGFR equation

**Table 4:** Relationship between clinico-radiological parameters and Uropathy/Nephropathy

	Uropathy (Freq (%)			Nephropathy (Freq (%)		
Variable	Yes(n=26)	No(n=88)	P-value	Yes(n=12)	No(n=102)	P-value
**Age Group**			0.370			0.431
20 – 29	2(13.3)	13(86.7)		0(00.0)	15(100.0)	
30 – 39	9(22.0)	32(78.0)		4(9.8)	37(90.2)	
40 – 49	12(23.1)	40(76.9)		7(13.5)	45(86.5)	
50 – 59	3(50.0)	3(50.0)		1(16.7)	5(83.3)	
**Uterine Size**			0.001*			0.002*
10/52 to 24/52	7(10.3)	61(89.7)		2(2.9)	66(97.1)	
25/52 to 39/52	19(41.3)	27(58.7)		10(21.7)	36(78.3)	
**Fibroid size**			0.13			0.015*
<5	23(21.3)	85(78.7)		09(8.3)	99(91.7)	
≥ 5	03(50.0)	03(50.0)		03(50.0)	03(50.0)	

*X^2^= Chi Square, DF= Degree of freedom*

* = *statistically significant*

**Table 5:** Binary Logistic Regression Uterine size, Fibroid size, Uropathy and Nephropathy

	Co-efficient	df	Sig.	Odd Ratio	95% C.I. for OR	
					Lower	Upper
Uropathy	2.938	1	.001	18.883	3.281	108.67
Uterine size	-1.092	1	.226	.335	.057	1.963
Fibroid size	2.051	1	.091	7.779	0.721	.8387

Key: **Independent variables** are uterine size, Fibroid size, Uropathy
